# MAP3K1 May be a Promising Susceptibility Gene for Type 2 Diabetes Mellitus in an Iranian Population

**Published:** 2016-08-21

**Authors:** Shahram Torkamandi, Milad Bastami, Hamid Ghaedi, Fateme Moghadam, Reza Mirfakhraie, Mir Davood Omrani

**Affiliations:** 1 *Department of Medical Genetics, Faculty of Medicine, Shahid Beheshti University of Medical Sciences, Tehran, Iran.*; 2 *Department of Medical Genetics, Faculty of Medicine, Tabriz University of Medical Sciences, Tabriz, Iran.*; 3 *Imam Hossein Hospital, Shahid Beheshti University of Medical Sciences, Tehran, Iran.*; 4 *Department of Medical Genetics, Faculty of Medicine, Shahid Beheshti University of Medical Sciences, Tehran, Iran.*

**Keywords:** Type 2 diabetes mellitus, genome- wide association study, *MAP*_*3*_*K*_*1*_, cytokines

## Abstract

Considering that MAPK (mitogen- activated protein kinase) signaling pathway has an important role in the progression of inflammatory cytokine secretion in type 2 diabetes mellitus (T2DM), we have recently investigated the reported genetic polymorphism from genome wide association study in *MAP*_3_*K*_1_ (mitogen-activated protein kinase kinase kinase 1) in diabetes as an important member of MAPK signaling. This study aimed to investigate the possible association of rs10461617 at the upstream of *MAP*_3_*K*_1_ gene in an Iranian case-control study with the risk of T2DM. The study population was comprised of 342 unrelated Iranian individuals including 177 patients with T2DM and 165 unrelated healthy control subjects. Genotyping was performed using PCR-RFLP and confirmed with sequencing. In a logistic regression analysis, the rs10461617A allele was associated with a significantly higher risk of T2DM assuming the log- additive model (OR: 1.44, 95% CI: 1.01-2.05, P = 0.039). In conclusion, we provided the first evidence for the association of rs10461617 at the upstream of *MAP*_3_*K*_1_ with the risk of T2DM in an Iranian population.

*Modern lifestyle, with abundant nutrient supply, high calorie dietary habits, and paucity of physical activity has resulted in a dramatic increase in the rates of metabolic-associated diseases including type 2 diabetes mellitus (T2DM) (* 1 *). T2DM is a heterogeneous disorder caused by complex interplay between genetics and environmental factors and is a substantial worldwide health problem which according to the International Diabetes Federation (IDF) more than 640 million of people worldwide will suffer from diabetes by 2040 (* 2 *).* *Critical pathology of T2DM results from insulin resistance and failure of pancreatic islet beta cells to increase insulin secretion due to gradual loss of beta cell mass and apoptosis (compensatory failure) (* 3 *). Indeed, only individuals with insulin resistance that are unable to do beta cell compensatory response develop progressive T2DM (* 4 *). Chronic hyperglycemia after beta cell failure trigger apoptosis of beta cell through inflammatory stress and cytokine secretion, endoplasmic reticulum stress, mitochondrial dysfunction and impairment of other important cellular hemostatic pathways (* 5 *-* 7 *). Genome wide association studies (GWASs) have transformed our knowledge of T2DM genetics and identified susceptibility of loci in molecular pathways that lead to the loss of beta cell mass and functions (* 8 *). Among these, MAPKs are the family of kinases and one of the most important pathways that transduce external signal to the nucleus in response to environmental stress resulting from hyperglycemia (* 9 *). Few and inconsistent reports of MAPK pathway are available for T2DM but the results indicate that this pathway may be altered. In addition, different studies have explored the dysregulation of MAPK pathway members including ERK1/2, MEK1, JNK1 and p38-MAPK in the basal state and the effects on insulin pathway in T2DM patients compared to control subjects (* 10 *-* 13 *). MAP*_3_ *K*_1_ *consists of twenty coding exons and encodes a 164 kDa serine/threonine kinase and is a member of different signal transduction cascade of MAPK, including the ERK, JNK and p38-MAPK kinase. MAP*_3_ *K*_1_ *is also interacting with insulin pathway (* 13 *). The association of* *MAP*_3_ *K*_1_ *with the risk of T2DM has been shown recently with GWASs (* 14 *).* *To the best of our knowledge, there are no data regarding the possible contribution of the GWAS- identified gene locus to T2D M and therefore, in this study we evaluated the association of* *MAP*_3_ *K*_1_ *rs10461617 G>A, an index variant of the GWAS identified locus with the risk of T2DM in an Iranian population.*

## Materials and methods

**Subjects**

The study population was composed of 342 unrelated Iranian individuals, including 177 patients with T2DM and 165 unrelated healthy control subjects who were defined based on the WHO criteria and matched for age and gender (15, 16) (Table 1). T2DM was diagnosed as fasting plasma glucose (FPG) levels of ≥ 126 mg/dl and 2-hour glucose concentrations of ≥ 200 mg/dl after a 75 g oral glucose tolerance test or HbA_1_C> 6.5 %. Written informed consent was obtained from all subjects of this study which was approved by the Ethics Committee of Shahid Beheshti University of Medical Sciences. The control subjects enrolled were those without any past documented history of glucose intolerance or family history of diabetes and had fasting plasma glucose concentrations below 100 mg/dl.

**Genotyping and DNA extraction**

Genomic DNA of the subjects was extracted from peripheral blood leukocytes following a standard salting out protocol. Genotyping of rs10461617 of *MAP*_3_*K*_1_ gene was carried out using polymerase chain reaction in 25 µl of reaction mixture and followed by restriction fragment length polymorphism (PCR-RFLP) analysis. The PCR cycling conditions for rs10461617 analysis as well as amplicon size and restriction fragments obtained after HinfI digestion are indicated in Table 2. Primer 3 was used for designing primers. rs10461617 is located upstream of *MAP*_3_*K*_1 _(chromosome 5, hg38 SNP position 5680848) (17). To confirm the validity of the genotyping method, 20 samples were randomly selected and sequenced after DNA amplification.

**Statistical analysis**

Fisher's exact test was applied for significant departure from Hardy-Weinberg equilibrium among patients’ and controls’genotyping and all statistical analyses were performed by R programming language (version 3.1.0) (18). Additionally, student *t*-test was applied for evaluating differences in clinical variables and demographic characteristics between the patients and controls and Pearson’s χ^2 ^test was used for categorical variables. Multivariate logistic regression analysis was performed to control age, sex and BMI category. The association of rs10461617 with T2DM was evaluated using logistic regression analysis which was implemented in the SNPassoc package (version 1.9-2) and distribution of genotype frequencies was analyzed under five genetic models (codominant, dominant, recessive, overdominant and log additive). Odds ratios (OR) together with 95% confidence intervals (95% CI) were calculated and a p-value< 0.05 was considered to be statistically significant in this study.

## Results

**Population characteristics**

Table 1 shows the subjects’ clinical characteristics and demographic data. The patients and controls in this study were matched for age and sex. According to the results, the patients had higher levels of BMI and FPG than those of the controls.

**Table 1:** Characteristics of the study population

	Patients (n= 177)	Controls (n= 165)	P value
Age (years)	59.93 ± 11	59.65 ± 9.05	0.7932
Sex, Male (%)	43.69	46.44	-
BMI (Kg/m^2^)	31.26 ± 5.02	25.97± 1.69	< 2.2e-16
FPG (mg/dl)	148.27 ± 27.43	88.47 ± 9.57	< 2.2e-16

**Table 2:** The primers sequences of RFLP, sequencing and PCR conditions for the rs10461617 genotyping

SNP	Type of primers	Primers sequences 5' → 3'	PCR condition ( ^o^C/s )			Amplicon/ fragment size (bp)
			Denaturation	Annealing	Extension	
rs10461617	PCR-RFLP	F:GC**A**CAGCTTCACcATGCCTTGgggggggg	95/30	63/30	72/30	117/99+18
		R:CCTGTGAGGTCCTCCCTG**A**GT				
	Sequencing	F:AAACGAAATGGTCTCTGCTCCAG	95/30	61/30	72/30	702
		R:GGTTCAAGAGCCACATAGTTGCT				

**Association of rs10461617** **polymorphism with T2DM**

The genotype frequencies of rs10461617 are presented in Table 3 which were not significantly deviated from Hardy–Weinberg equilibrium among the controls (P value: 0.22). The distribution of genotype frequencies in different modes of inheritance for rs1046167 in the patients and the controls is presented in Table 4. We found that *MAP*_3_*K*_1_ rs10461617 was associated with increased risk of T2DM assuming log-additive modes of inheritance (OR: 1.44, CI: 1.01- 2.05, P = 0.039) (Table 3).

**Table 3:** Genotypes frequencies of rs10461617 in studied population

	All subjects n=342		Patients n=177		Controls n=165	
Genotype	Count	Proportion	Count	Proportion	Count	Proportion
AA	22	0.06	8	0.05	14	0.08
GA	106	0.31	50	0.28	56	0.34
GG	214	0.63	119	0.67	95	0.58

**Table 4:** The distribution of genotypes in the T2DM cases and controls

**Model**	**Genotypes**	**Patients** **N (%)**	**Controls** **N (%)**	**OR** **(95% CI)**	**P** **value**	**AIC**	**BIC**
Codominant	GG AG AA	119 (67.2) 50 (28.2) 8 (4.5)	95 (57.6) 56 (33.9) 14 (8.5)	1.00 1.40 (0.882.24) 2.19 (0.885.44)	0.1222	475.44	486.99
Dominant	GG	119 (32.8)	95 (57.6)	1.00	0.065	474.3	482
	AG+AA	58 (54.1)	70 (42.4)	1.51 ( 0.97-2.35)			
Recessive	GG+AG	169 ( 95.5)	151 (91.5)	1.00	0.13	475.4	483.1
	AA	8 (4.5)	14 (8.5)	1.96 (0.80 – 4.80)			
Overdominant	GG+AA	127( 71.8)	109 (66.1)	1.00	0.26	476.4	484.1
	AG	50 (28.2)	56 (33.9)	1.30 ( 0.82 –2.07)			
log- Additive	-	-	-	1.44 (1.01-2.05)	**0.039**	473.5	481.1

CI: confidence interval; OR: odds ratio; *AIC*: *Akaike information criterion; BIC: Bayesian information criterion;* P value for the most probable genetic models is indicated with bold face

## Discussion

Completion of the human genome sequence led to advances in medical research due to detailed maps of common single nucleotide polymorphisms (SNPs) location (19). After that, GWASs as a “hypothesis-free” approach and SNP- arrays, have opened new areas of molecular genetics to dissect the phenotypic variation of T2DM into individual genetic variants and led to the discovery of more than seventy entirely new T2DM loci (20, 21). Each of these discovered genes and loci, exert a small effect, whether individually or interactively acting with different complex pathways. Therefore, genetic analysis of a multifactorial and complex disease such as T2DM is very complicated (22). Given that SNPs of GWASs were used as “tag” markers of functional variants located at that haplotype, the next major step is to evaluate other linked SNPs to find strongest variants and finally identify causal genes (23). In other words, due to linkage disequilibrium between particular alleles at each haplotype, any SNP at defined loci, tend to be co-inherited and as such it is needed to define causal variants at each proposed GWAS locus with fine-mapping in different ethnicities and functional studies (24). While it is known that T2DM is an inherited disease, the heritability of GWAS variants that are primarily derived from European ancestry studies, is still obscure in Iranian population. Among these, a variance in the upstream of *MAP*_3_*K*_1_ has been shown to modify the susceptibility to T2DM. In a recent GWAS study also, the association of rs10461617 with the risk of T2DM was observed in DIAGRAM+ and Dravidians (14). To the best of our knowledge, there are no data regarding the possible contribution of the GWAS-identified locus at *MAP*_3_*K*_1_ to T2DM in an Iranian population. We showed that rs10461617 is significantly associated with T2DM in additive model in our population. In other words, each copy of risk allele modifies the risk in additive form in comparison with other forms of inheritance and AA homozygous genotype has a double risk than heterozygous AG genotype. Moreover, this is the first replication study for this SNP and the results were in line with the original GWAS and DIAGRAM+with the A allele being associated with an increased risk of T2DM. Rs10461617 is located at the 5’ upstream of *MAP*_3_*K*_1_*,* Many lines of evidence demonstrated that variants located at the 5’ upstream regulatory elements, can affect the quantitative trait of neighboring genes (25)*. *MAP_3_K_1 _is a serine/threonine kinase and a member of MAPK signal transduction cascade which phosphorylates and activates MAPKs, ERK, JNK, p38 in the next step (13). This signal transduction also affects insulin pathway in beta cells to regulate blood glucose levels (26). In addition, MAPK and their downstream targets have pivotal role in cellular response to environmental stress during hyperglycemia (27). It is widely accepted that hyperglycemia stress, increases cytokines secretion such as TNF-α, IL-6 and IL-1β and alters gene expression profile in targeted cells (28-30). Oetjen et al. illustrated that IL-1β through the use of MAP_3_K_1_ prevents insulin gene transcription and suggested that inhibition of MAP_3_K_1_ reduces the progression from prediabetic to diabetes mellitus state (31). It has also been reported that in vitro overexpression of MAP_3_K_1_ induces JNK phosphorylation through cytokine mediated pathway and leads to stress-induced beta cell death (13). The interesting relevance of MAPK signaling and inflammatory cytokine secretion and insulin resistance was also studied in skeletal muscle, adipocytes, retinal and hepatic tissues of T2DM patients and models (11, 32-34). Walker et al. identified increased stress kinase p38-MAPK as a downstream of MAP_3_K_1_ and this is a key proinflammatory-induced regulator in skeletal muscle. They also treated cells with p38-MAPK inhibitor and observed reduced cytokines secretion (34). The activation of MAPK proteins in hepatic cells resulted from hyperglycemia and inflammatory stimuli, increased insulin receptor phosphorylation and insulin resistance in mice model of diabetes (33, 35, 36). Therefore, hyperglycemia and stress stimuli have been strongly associated with increased inflammatory element secretion, insulin resistance and beta cell death. MAPK signal transduction components have been suggested as important candidates for these pathogeneses in T2DM and in vitro studies demonstrated improvement of inflammation stress with MAPK inhibitors (34). In this context, SNPs and genomic variants at this locus may have important effects on the quantity and quality of MAP_3_K_1_ and association with diabetic pathogenesis. Therefore, MAP_3_K_1_ is a member of MAPK signal transduction in response to stress stimuli of hyperglycemia, and genomic variation at this gene may have important roles in beta cell death and insulin resistance and inflammatory cytokine secretion.

In conclusion, rs10461617 a SNP located upstream of MAP3K1 is significantly associated with T2DM in our population. This is the first replication study for this SNP and the results were in line with the original GWAS and DIAGRAM+ with the A allele being associated with an increased risk of T2DM, although one of the limitations of this study was the small sample size. It is then suggested to replicate this SNP and other variants at this locus in different populations with focusing on phenotype and insulin levels to determine functional variants.
